# Effects of the Sintering Process on Nacre-Derived Hydroxyapatite Scaffolds for Bone Engineering

**DOI:** 10.3390/molecules25143129

**Published:** 2020-07-08

**Authors:** Rohaya Megat Abdul Wahab, Nurmimie Abdullah, Shahrul Hisham Zainal Ariffin, Che Azurahanim Che Abdullah, Farinawati Yazid

**Affiliations:** 1 Department of Family Oral Health, Faculty of Dentistry, Universiti Kebangsaan Malaysia, Jalan Raja Muda Abdul Aziz, Kuala Lumpur 50300, Malaysia; rohaya_megat@ukm.edu.my (R.M.A.W.); drnurmimie@gmail.com (N.A.); 2 Centre for Biotechnology and Functional Food, Faculty of Science and Technology, Universiti Kebangsaan Malaysia, Bangi 43600, Selangor, Malaysia; shahroy8@gmail.com; 3 Department of Physics, Faculty of Science, Universiti Putra Malaysia, Seri Kembangan 43400, Selangor, Malaysia; azurahanim@upm.edu.my

**Keywords:** hydroxyapatite, nacre, marine scaffold, bone engineering

## Abstract

A hydroxyapatite scaffold is a suitable biomaterial for bone tissue engineering due to its chemical component which mimics native bone. Electronic states which present on the surface of hydroxyapatite have the potential to be used to promote the adsorption or transduction of biomolecules such as protein or DNA. This study aimed to compare the morphology and bioactivity of sinter and nonsinter marine-based hydroxyapatite scaffolds. Field emission scanning electron microscopy (FESEM) and micro-computed tomography (microCT) were used to characterize the morphology of both scaffolds. Scaffolds were co-cultured with 5 × 10^4^/cm^2^ of MC3T3-E1 preosteoblast cells for 7, 14, and 21 days. FESEM was used to observe the cell morphology, and MTT and alkaline phosphatase (ALP) assays were conducted to determine the cell viability and differentiation capacity of cells on both scaffolds. Real-time polymerase chain reaction (rtPCR) was used to identify the expression of osteoblast markers. The sinter scaffold had a porous microstructure with the presence of interconnected pores as compared with the nonsinter scaffold. This sinter scaffold also significantly supported viability and differentiation of the MC3T3-E1 preosteoblast cells (*p* < 0.05). The marked expression of Col1α1 and osteocalcin (OCN) osteoblast markers were also observed after 14 days of incubation (*p* < 0.05). The sinter scaffold supported attachment, viability, and differentiation of preosteoblast cells. Hence, sinter hydroxyapatite scaffold from nacreous layer is a promising biomaterial for bone tissue engineering.

## 1. Introduction

Bone defects from trauma, as well as congenital and pathological disease are areas of concern in dentistry. There are various treatment modalities commonly practiced including bone grafts [1]. There are a few bone grafts available such as autograft, allograft, xenograft, and bone substitutes.

Autograft is harvesting bone from one site and transplanting to another site of the body in the same individual. Despite various options available, autograft is still considered to be the “gold standard” for treatment of bone defects with reported success rates as high as 95% [2]. Since the bone is harvested from the same patient, the risk of immunoreaction and disease transmission are minimal [1]. However, autograft requires a second surgical site which is the main drawback of this graft. Children are far more affected by this treatment option as they can suffer from complications after surgery which indirectly affects their quality of life [2,3]. Allograft involves harvesting bone from a different person other than the receiver. The use of allograft alleviates the need for an additional surgical site which in turn reduces the donor site pain and morbidity. Nevertheless, the main concerns with allograft are immunoreaction and disease transmission [4]. Protein deactivation is possible in order to keep the risk at a minimal level, but this technology imposes a significant high cost for the material. Xenograft which is bone graft harvested from different individuals and species are among the choices available. Bio-Oss^®^ is the most commonly used xenograft in the maxillofacial region [5]. Although it is an inexpensive option as compared with allograft, this graft exhibits poor vascularity and mechanical property for use in critical-sized defects [6]. Furthermore, the application is also limited by a patient’s religion and belief [4].

With the advancement of tissue engineering, bone tissue regeneration is a promising way to treat bone defects. The triad of cells, scaffolds, and growth factors are essential components of tissue engineering. Hydroxyapatite, a bioceramic scaffold commonly used in bone regeneration due to its similar chemical components with native bones can be derived naturally or synthetically. In fact, hydroxyapatite from natural origin has been reported to have no stoichiometric elements [7]. There are various natural sources for hydroxyapatite scaffolds which include deproteinized bovine bone, porcine bones, eggshell waste, and woods [8]. In addition, marine sources offers a wide range of natural sources for extracting hydroxyapatite such as coral, bone fish, fish scales, seashells, nacres, and cuttlefish bones [7,8,9,10,11,12]. As the world population increases, the waste from consumption of seafood delicacies also increases. Silva et al. [13] reported that approximately three million tons of oyster shell wastes were discarded each year. Hence, proper disposal of this biologic waste is crucial in order to prevent contamination to both land and marine sources.

The nacreous layer, which is the inner layer of bivalve shells, such as oyster and mussels, mainly consists of an aragonite layer [14,15]. The ”brick and mortar” arrangement of the aragonite layer contributes to its ability to prevent crack propagation and deformation in response to mechanical insults [16]. The superior mechanical property of the nacreous layer is a promising hallmark as a biomaterial for bone regeneration. Inorganic aragonite tablets are predominantly calcium carbonate, homologous to calcium phosphate (hydroxyapatite) in human bone [16]. There are several methods for transforming the nacreous layer into hydroxyapatite which include sintering, wet chemical precipitation, hydrothermal, sol-gel, and ball-milling [8,11,17,18]. Wet chemical precipitation, hydrothermal, and sol-gel methods have mostly been used for the application of hydroxyapatite as a coating material on the surface of a metallic implant or scaffold [17,19]. This type of combination overcomes the weakness of hydroxyapatite, as well as metal scaffolds, by enhancing the mechanical properties and corrosion resistance and, at the same time, promotes bone-bonding ability [19]. Meanwhile, the sintering process involves formation of a solid mass using heat without melting it to the point of liquefaction [10]. The sintering process has also been employed to improve the mechanical properties of hydroxyapatite scaffold. Microstructural properties such as crystallinity, grain size, density, and microporosity greatly influence the mechanical performance of hydroxyapatite scaffold. These microstructural properties can be controlled by modifying the sintering temperature. Better bioactivity for hydroxyapatite scaffold can be achieved using a lower sintering temperature, whereas better mechanical properties can be achieved using a higher sintering temperature [17]. In this study, a higher sintering temperature was used during the fabrication of the sinter hydroxyapatite scaffold.

To date, the use of inner molluscan shells is a relatively underexplored biomaterial, particularly, in dental applications. The reusing of shell waste as biomaterial for bone tissue regeneration could potentially be used as an alternative to bone graft and would indirectly contribute to a green environment. Previous studies have assessed the effect of nacre hydroxyapatite scaffold and have basically emphasized the biological, chemical, physical, and regeneration properties of the scaffold, or the combined effect of these properties [17,20,21,22]. The effect of the sintering process on nacre hydroxyapatite scaffold for in vitro physical and osteogenic potentials has not been well documented. Therefore, we decided to compare the in vitro effect between sinter and nonsinter hydroxyapatite scaffolds from the nacre layer of mollusc species as a potential biomaterial for bone engineering in dentistry. The objectives were (i) to characterize the physical properties of both sinter and nonsinter scaffolds and (ii) to determine the osteogenic potential between both sinter and nonsinter hydroxyapatite scaffolds.

## 2. Results

### 2.1. Characterization of Hydroxyapatite Scaffolds

#### 2.1.1. Pore Size

**Figure 1:**
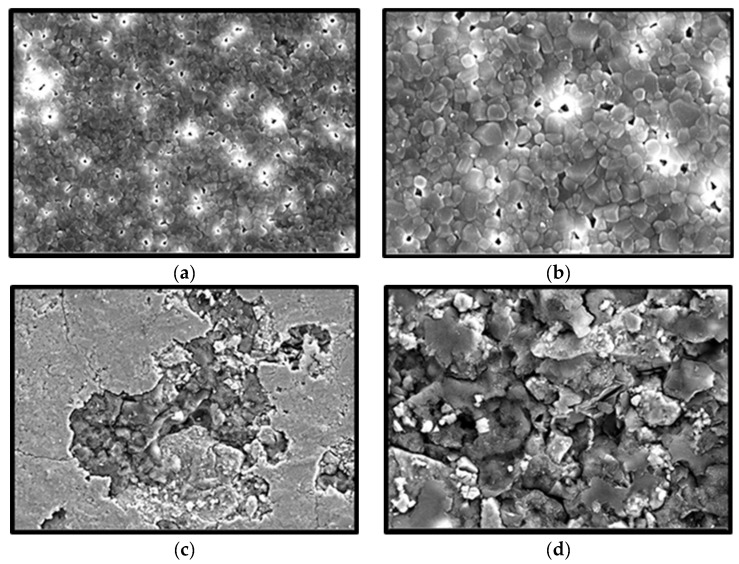
Field emission scanning electron microscopy (FESEM) images of hydroxyapatite scaffolds. Sinter scaffolds (**a**) 100× magnification; (**b**) 1000× magnification. Nonsinter scaffolds (**c**) 100× magnification; (**d**) 1000× magnification.

The field emission scanning electron microscopy (FESEM) micrographs of sinter and nonsinter hydroxyapatite scaffolds are represented in Figure 1. The sinter scaffolds are composed of multiple polygonal-shaped particles. The presence of irregular micropores scattered in between the particles appears as a black halo. At a higher magnification, as shown in Figure 1b, the micropores were measured and the average pore size was 308 μm. Figure 1c,d shows the FESEM micrographs of the nonsinter hydroxyapatite scaffolds. These scaffolds have a compact structure and some pores are unevenly distributed. The pore size was difficult to measure.

#### 2.1.2. Composition

**Figure 2:**
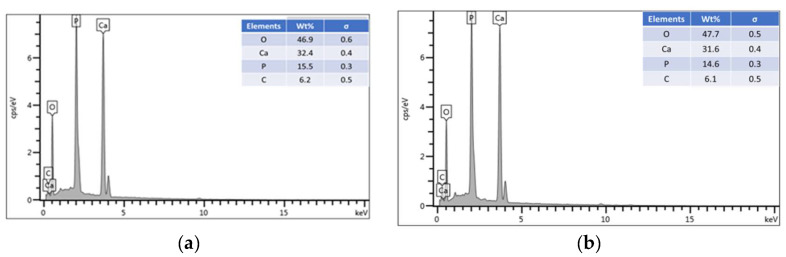
EDX analysis of sinter (**a**) and nonsinter (**b**) hydroxyapatite scaffolds.

The energy dispersive X-ray (EDX) analysis was conducted for the sinter and nonsinter scaffolds and the results are shown in Figure 2. The constituents are identified by the peaks with respect to their energy levels. Calcium (Ca) and phosphate (P) can be observed at higher peaks in both scaffolds. Moreover, other basic elements such as carbon (C) and oxygen (O) can also be observed.

#### 2.1.3. Porosity

Porosity and particle size of both sinter and nonsinter scaffolds were analyzed from the SEM-EDX analysis and images of micro-computed tomography (microCT) using a CTAn (Bruker, Belgium) analysis software. The average particle size for both scaffolds is 3.92 ± 0.62 µm. This indicates that the particle size of these hydroxyapatite scaffolds is small due to the high carbonate content which can inhibit the growth of crystal. For the sinter scaffolds, the functional porosity was approximately 20%, whereas the porosity of the nonsinter scaffolds was less than 5%. In addition, in the radiographic image, the nonsinter scaffolds appear to be denser than the sinter scaffolds.

### 2.2. Characterization of Hydroxyapatite Scaffolds Cell Culture Experiment

#### 2.2.1. Morphology of Preosteoblast Cell-Seeded Scaffolds

**Figure 3:**
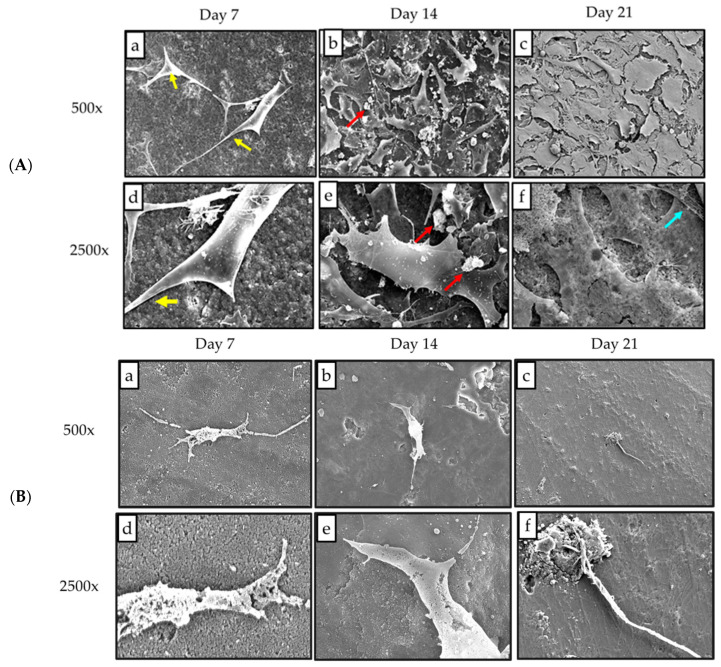
(**A**) FESEM micrographs of MC3T3-E1 preosteoblast cells seeded on the sinter hydroxyapatite scaffold. The presence of cytoplasmic extension (yellow arrow), hydroxyapatite nodules (red arrow), and fiber strands (blue arrow) were observed in both low (500×) and high magnification (2500×); (**B**) FESEM micrographs of MC3T3-E1 preosteoblast cells seeded on the nonsinter hydroxyapatite scaffolds. At higher magnification (2500×), cells exhibit destruction of cell morphology with absence of apatite nodules.

The FESEM micrographs of the MC3T3-E1 preosteoblast cells seeded on both sinter and nonsinter scaffolds are shown in Figure 3A,B. At the lower magnification (500x), the FESEM micrograph reveals that the number of cells on the surface of the sinter scaffolds increased continuously from day seven until day 14 (Figure 3Aa,b). The preosteoblast cells spread effectively and eventually covered the whole surface by day 21 (Figure 3Ac).

Moreover, the morphological changes of the MC3T3-E1 preosteoblast cells were observed at higher magnification (2500×) of FESEM. The preosteoblast cells appeared as elongated cells with finger-like projection of cytoplasmic extensions by day seven (Figure 3Ad). On day 14, the MC3T3-E1 preosteoblast cells proliferated further, and then changed into irregular-shaped osteocytes. The presence of apatite nodules was also observed adjacent to the osteocytes (Figure 3Ae). The morphology of preosteoblast cells observed on day 21 was polygonal in shape. Collagen fibers also appeared as multiple linear strands next to the cells, as indicated in Figure 3Af.

There was no increase in the number of cells observed from day seven until day 21, on the nonsinter scaffolds at a lower magnification of 500x (Figure 3B). On days seven and 14, the preosteoblast cells did not maintain their morphological structure after incubation (Figure 3Bd,e). The micrograph also exhibited the destruction of cell membranes of preosteoblast cells seeded on the nonsinter scaffolds. There was no evidence of apatite nodule and collagen fibers observed on the nonsinter scaffolds surface by day 21 (Figure 3Bf).

#### 2.2.2. Cell Viability

**Figure 4:**
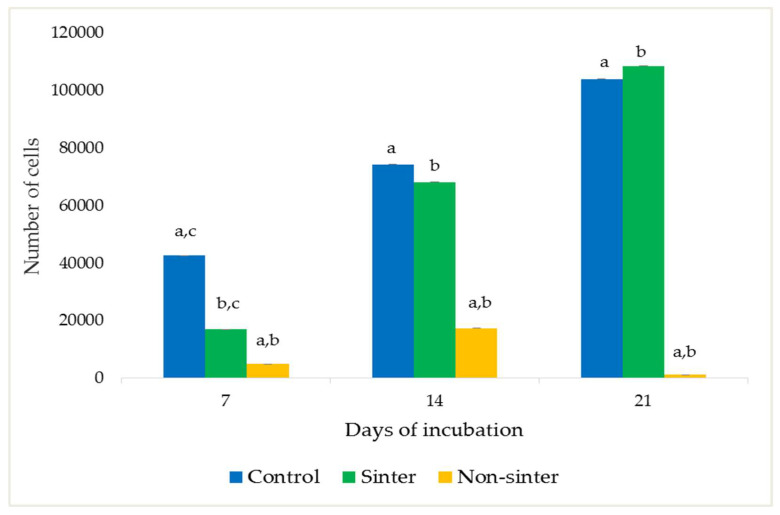
Viability of MC3T3-E1 preosteoblast cells seeded on sinter and nonsinter hydroxyapatite scaffolds. Cells without a scaffold were considered to be the control group. Same letter of a, b, c in each incubation day denotes significant differences within group (*p* < 0.05).

The viability of the MC3T3-E1 preosteoblast cells seeded on both the sinter and nonsinter hydroxyapatite scaffolds using MTT assay are illustrated in Figure 4. Cells without a scaffold were treated as the control group.

Regarding the sinter hydroxyapatite scaffolds, the mean number of cells was 16,888 cells after seven days of culture period. The mean number of MC3T3-E1 preosteoblast cells was statistically increased (*p* < 0.05) to 68,110 by day 14. On day 21 of incubation, the cell numbers were highly elevated to 108,555 cells, and were statistically significant (*p* < 0.05).

At seven days post seeding of the MC3T3-E1 preosteoblast cells on the nonsinter scaffolds, the mean cell number was 4888 cells. The number of cells increased on day 14 and decreased by the end of 21 days, after in vitro culture. However, there was no significant difference of cell numbers between all three days of observation in the nonsinter scaffolds.

The comparison of cell viability between the sinter and nonsinter scaffolds showed that the number of preosteoblast cells on the sinter scaffolds was significantly higher (*p* < 0.05) on day seven and 14 after incubation. The difference between the number of viable cells on both scaffolds, by day 21, was also remarkable (*p* < 0.05).

#### 2.2.3. Cell Differentiation

**Figure 5:**
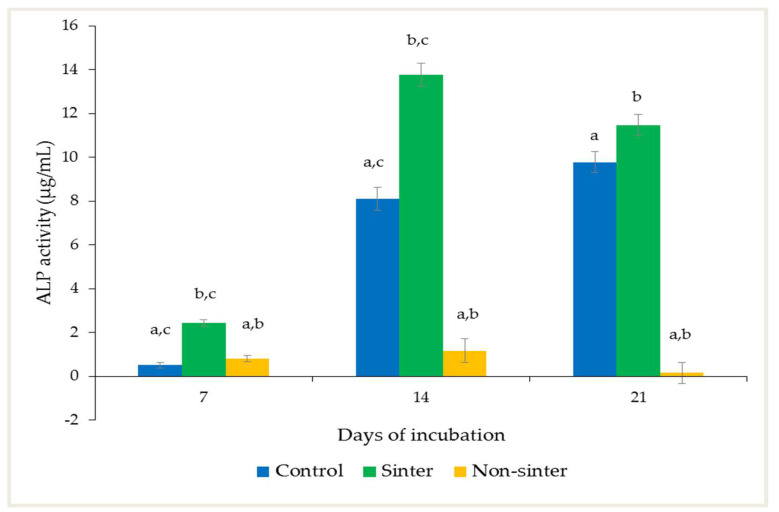
Alkaline phosphatase (ALP) activity of MC3T3-E1 preosteoblast cells seeded on sinter and nonsinter hydroxyapatite scaffolds. Cells without a scaffold were considered to be the control group. Same letter of a, b, c in each incubation day denotes significant differences within group (*p* < 0.05).

The alkaline phosphatase (ALP) activity of the MC3T3-E1 preosteoblast cells seeded as the control group, and the sinter and nonsinter hydroxyapatite scaffolds at different incubation periods are shown in Figure 5.

The mean expression of the ALP activity from the MC3T3-E1 preosteoblast cells seeded on the sinter scaffolds by day seven was 2.45 μg/mL. The highly elevated ALP activity was significant after 14 days of observation (*p* < 0.05). By day 21, the expression was decreased, but the difference was not statistically significant.

The ALP activity of the MC3T3-E1 preosteoblast cells seeded on the nonsinter scaffolds was 0.82 µg/mL after seven days of incubation. The expression showed a significant increment from day seven until day 14 of culture (0.210 ± 0.042) (*p* < 0.05). The preosteoblast cells showed a decreasing trend of ALP activity by day 21. However, the difference of ALP activity between these days was not statistically significant.

A comparison within groups at each incubation period showed that the difference of ALP expression between the sinter and nonsinter scaffolds was significant (*p* < 0.05) on day seven. The high expressions of ALP activity by cells seeded for all three groups after 14 days of culture were also statistically significant (*p* < 0.05). After 21 days of incubation, the significantly higher ALP activity was observed in two comparison groups, i.e., the sinter versus nonsinter group and the nonsinter versus the control group (*p* < 0.05).

#### 2.2.4. Osteoblast Markers

In order to further confirm the osteogenic potential of both hydroxyapatite scaffolds, the expressions of osteoblastic markers on day 14 were analyzed using real-time polymerase chain reaction (rtPCR). The MC3T3-E1 preosteoblast cells without a scaffold were used as the control group. The 2ΔCT method was used by normalizing with Gapdh housekeeping gene expression and presented as relative expression to the control group.

**Figure 6:**
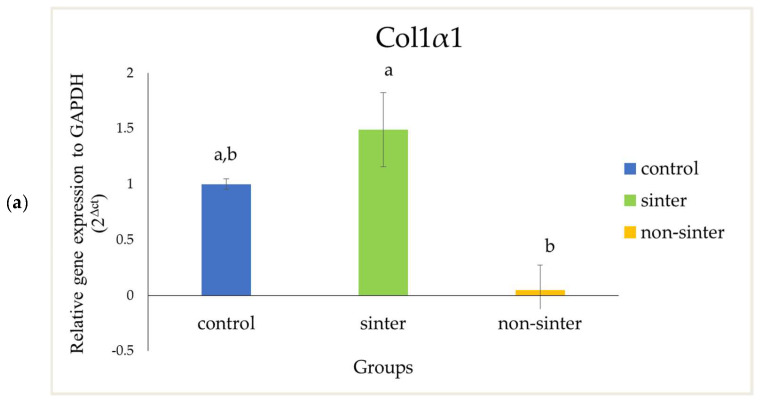

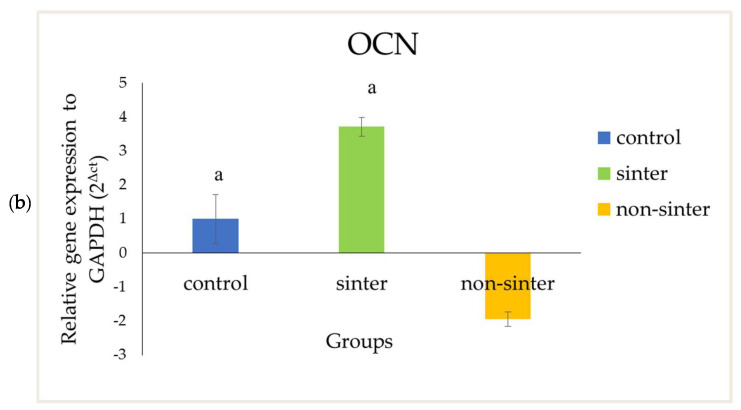
Relative expression of osteoblast markers (**a**) Colα1 and (**b**) osteocalcin (OCN) for cells seeded on sinter and nonsinter hydroxyapatite scaffolds, and the control group. Same letter of a, b denotes significant differences within group (*p* > 0.05).

The relative expression of Col1α1 by the preosteoblast cells is illustrated in Figure 6 (top). In the sinter scaffolds, Col1α1 was highly expressed as compared with the control group, after 14 days of incubation. However, there was only a 0.05-fold change of expression in the nonsinter scaffolds as compared with the control group.

Real-time PCR of the osteocalcin (OCN) osteoblast marker is illustrated in Figure 6 (bottom). The expression of OCN by the MC3T3-E1 preosteoblast cells seeded on the sinter scaffolds was significantly upregulated to 3.71-fold as compared with the control group. Meanwhile, the expression of OCN was downregulated in the nonsinter scaffolds (*p* < 0.05).

## 3. Discussion

The growing human population will indirectly increase biogenic waste discarded from the food industry every year. Processing and recycling the remaining waste such as scales, fish bones, and seashells would enhance the value added for these by-products as a result of minimizing the undesirable environmental impact and also offering a great alternative as a biomaterial in medical applications. Nacre, from the inner shell layer of the mollusc species is a promising biomaterial for bone regeneration. The observed polygonal shaped microstructure and ~300 µm pore size of sinter hydroxyapatite, in the present study, was similar to those previously reported from nacre origin and aragonite structure of the mollusc species [16,23,24]. On the contrary, it is difficult to measure the pore size of nonsinter scaffold because the particles are very dense and are arranged in a haphazard manner. Therefore, the sintering process has a crucial effect on the pore size, density, and mechanical properties of hydroxyapatite [25].

The EDX analysis showed the presence of similar elements between both scaffolds with a slightly lower percentage value in the nonsinter scaffolds. The presence of elements such as calcium (Ca), phosphorous (P), and oxygen (O) are comparable to previous findings of hydroxyapatite transformation from biogenic marine waste via the sintering process [10]. The finding that the inorganic composition of nacre, mainly calcium and phosphate, are relatively similar to natural bone is in agreement with previous studies, and therefore, potentially, it can be used as a natural biomaterial in bone regeneration [16,23,26]. This remarkable finding also suggests that the sintering process does not affect the inorganic component of nacre but could alter the microproperties of scaffolds such as morphology of aragonite tablets and presence of pore.

Total porosity is a measure of the voids in the scaffolds. The microCT analysis demonstrated that the functional porosity of the sinter group was approximately 20%. This finding was in contrast with 55% to 74% porosity suggested for bone regeneration by Bose et al. [27]. However, this conclusion was made from a previous study which utilized conventional methods that were less accurate as compared with the quantitative analysis with microCT. In addition, the nonsinter scaffolds had porosity less than 5% which were considered to be dense biomaterials according to Natasha et al. [28].

After initial seeding of cell lines, scaffolds were transferred into a new well to prevent false positive readings from cells that adhered to the side of wall. This explains the fact that the number of cells on the sinter scaffolds are much fewer as compared with the control group, one-week post incubation. Nonetheless, the results of the MTT assay demonstrated that the sinter scaffolds enhanced proliferation of preosteoblast cells after 7, 14, and 21 days of incubation. Cells on the sinter scaffolds were significantly increased on day 14. This finding was similar to a previous report on hydroxyapatite scaffolds [29]. The number of cells on the sinter scaffolds was also considerably higher than the control group at the end of 21 days, which demonstrated the osteoconductivity of these scaffolds. This finding suggests that sinter scaffolds support and enhance proliferation and viability of cell lines.

The sinter scaffolds also exhibited significantly better cell viability as compared with the nonsinter scaffolds. Such an observation showed good cytocompatibility and enhanced cell viability of sinter scaffolds with an incubation period. This result also supported the earlier FESEM findings of sinter and nonsinter scaffolds. The changes in cell morphology when cultured on sinter and nonsinter hydroxyapatite scaffolds are due to the presence of pores. Sinter scaffolds supports better cell morphology due to the presence of pores that provide good space for cells to proliferate. The open and interconnected pores in sinter scaffolds allow transportation of nutrients and metabolic waste which further translate with continuous proliferation and better cell growth [30]. Large pore size is suitable for cell attachment and penetration into a scaffold, whereas small pores provide a tunnel for cell metabolism [31]. To date, most researchers have reached a consensus that the optimum required pore size for bone regeneration ranges from 200 μm to 500 μm [32]. Our results indicated that the sinter scaffolds with an average pore size of about 308 μm were within the suggested range, and showed better cell attachment, morphology, and proliferation. Although some researchers have advocated that dense scaffolds also have comparable results, our findings did not support this argument [26]. Moreover, nonsinter scaffolds have very dense microstructure arrangements without pore structure, which portrays the destruction of cell morphology and lack of cell growth regardless of the culture time. This observation was depicted by a significant lower number of cells in the nonsinter scaffolds after 21 days of culture. The lack of pores in the nonsinter scaffolds hinders nutrient and metabolite transfer, and thus prevents cell growth with direct implications on cell morphology.

Osteoblast differentiation of scaffold can be evaluated by an increase in alkaline phosphatase (ALP) activity and mineralized nodule formation. The activity of ALP is used to indicate the presence of osteoblast cells and the formation of new bone. Nonetheless, ALP activity alone is insufficient to make a conclusion regarding the osteogenic capacity of cells. Hence, mineralization of cells on a scaffold should be determined using alizarin red staining and rt-PCR. However, in this study, mineralization of cells on the sinter and nonsinter hydroxyapatite scaffolds was not evaluated using alizarin red staining but by an upregulated expression of osteocalcin (OCN) marker. Other studies did not perform alizarin red staining but used rt-PCR to demonstrate the expression of OCN marker as an osteogenic maturation and occurrence of mineralization marker [33,34].

Alkaline phosphatase is an early marker of osteoblast differentiation [17]. In the control group, this marker was continuously elevated in a time-dependent manner. The consistent increase of ALP was related to the progressive differentiation of MC3T3-E1 preosteoblast cells. This result was in accordance with a previous study that reported the association of ALP expression with osteoblast differentiation cultured in medium supplemented with ascorbic acid and β-glycerophosphate [35].

Furthermore, the ALP expression of cells seeded on the sinter scaffolds peaked on day 14. While ALP production in the control group increased over time, the expression in the sinter scaffolds decreased after 21 days. MC3T3-E1 preosteoblast cells have been shown to express strong upregulation of ALP during cultivation and downregulate during osteoblasts maturation, as well as subsequent osteocytic differentiation [36]. This slight downregulation of ALP was postulated due to cells approaching the later stage of differentiation with the formation of mature bone.

Nevertheless, the expression of ALP in the nonsinter scaffolds significantly increased from day seven until day 14, although the expression was much less than the other groups. However, the activity on day 21 was not significant and almost diminished. It is possible that the nonsinter scaffolds only allowed cell attachment and differentiation until 14 days and caused cell death afterwards. This finding was also consistent with the earlier MTT assay of cells on the nonsinter scaffolds, in this study.

Taking into account the positive results of the MTT and ALP assays on day 14, our study showed that the sinter scaffold could potentially induce better differentiation and maturation of MC3T3-E1 preosteoblast cells as compared with the nonsinter scaffolds. The significant increase of ALP activity, in this present study, was not influenced by the incorporation of dexamethasone because this substance was omitted as the differentiation medium [36].

In this present study, the osteogenic differentiation related gene expression of OCN and Col1α1 have higher expression in the sinter scaffolds as compared with the nonsinter counterpart. However, the difference of Col1α1 between sinter and nonsinter scaffolds was not statistically significant (*p* > 0.05). The real-time polymerase chain reaction (rtPCR), in this present study, demonstrated higher expression of Col1α1 and significant marked upregulation of cells in the sinter scaffolds. These results support earlier findings of ALP expression and suggest the osteogenic potential of sinter scaffold in bone regeneration. Collagen type I (Col1α1) is always considered to be an early osteoblastic marker. The presence of this marker indicates cell adhesion and osteoblasts differentiation stage [36]. Meanwhile, osteocalcin (OCN) has been considered to be an important late osteogenic differentiation marker and a good marker for osteogenic maturation [33,34,35,37]. Another group of researchers considered OCN to be an early osteocyte marker [36,37]. Nonetheless, the result from this study showed marked expression of OCN on day 14. This finding was comparable with a study by Zhou et al. [34] where they found a significant increase of OCN levels with incorporation of hydroxyapatite coating after 14 days of treatment.

Liu et al. [31] found that incorporation of nacre onto scaffolds would stimulate better cell proliferation and alkaline phosphatase activity after two weeks of observation. Moreover, a study by Bianchi et al. [38] indicated the potential of coating or thin films hydroxyapatite for influencing cell morphology, proliferation, and osteoblast differentiation. Coating or thin films hydroxyapatite preserved the typical biological properties of cells and also improved their ability to osteogenic commitment [38]. Comparable to their results, our study reported similar positive results from SEM, MTT assay, and ALP activity. In addition, the extension of observation days, up to 21 days in the present study, was more valuable since the trend of osteoblastic differentiation was more clearly observed. It has been postulated that nacre contains natural growth factors including bone morphogenetic proteins (BMPs) that could influence cell adhesion, proliferation, and osteoblast differentiation [16,31,39]. The sintering process transforms the aragonite layer of nacre into hydroxyapatite [16]. Hydroxyapatite provides nucleating sites for precipitation of apatite crystals, which potentially consist of trace ions for more favorable biological effects [29,40]. The cell attachment, proliferation, and mineralization for sinter scaffolds, in our study, observed by FESEM micrographs correlated well with the proliferation assay and ALP activity results. These results are comparable to other published studies on hydroxyapatite [30,40]. Therefore, the attachment and proliferation of MC3T3-E1 preosteoblast cells on sinter scaffolds indicated the cytocompatibility of this biomaterial.

Moreover, the sintering process is also important for the microstructural properties of scaffold such as porosity, grain size, and pore size which determine the performance of the biomaterial [41]. In this study, the presence of pores in the sinter scaffolds as compared with the nonsinter scaffolds, as observed from the FESEM micrographs, suggested that the sintering process could influence formation of pores in the nacreous layer of molluscan shells [26]. However, these findings were in contrast with a previous study that suggested the sintering process by temperature was unsuitable for nacre due to the possible loss of protein during the procedure [23]. Although the porosity in the sinter scaffolds was approximately 20%, we postulated that interconnected pore size was more crucial for cell viability. The decreased porosity of the sinter scaffolds indirectly increased the mechanical property of these scaffolds. Thus, these scaffolds are potentially suitable as biomaterials for regeneration of bone defect.

We postulate that sinter hydroxyapatite scaffolds hastened the osteogenic differentiation of MC3T3-E1 preosteoblast cells because the maturation stage was taking place earlier, i.e., by day 14. The marked high expression of ALP activity and OCN on day 14 demonstrated that the sintering process of hydroxyapatite from the nacre layer could efficiently promote differentiation and maturation of MC3T3-E1 preosteoblast cells. Hence, our study suggests that the sintering process could be a possible way to enhance the property of nacre scaffolds for bone regeneration.

## 4. Materials and Methods

### 4.1. Fabrication of Hydroxyapatite Scaffolds

Hydroxyapatite scaffolds derived from the nacre layer were obtained from the Department of Physics, Faculty of Science, Universiti Putra Malaysia (UPM). The spherical-shaped scaffolds measuring 12 × 5 mm were provided in two groups, i.e., sinter and nonsinter scaffolds. Briefly, the nacre shells were cleaned and dried in an oven at 300 °C for an hour. Then, the nacre powder was calcined in an electric furnace for 2 h at 1000 °C and finely crushed with mortar and pestle. Calcium oxide derived from the nacre powder was used to prepare 0.5 M of calcium hydroxide solution. Following that, 0.3 M phosphoric acid was dropped wisely into the calcium hydroxide solution while stirring using a magnetic stirrer with speed 400 rpm and temperature at 40 °C. A few drops of ammonia solution were added into the mixture to adjust the pH value to 9 with continuous stirring for one hour. The gelatinous precipitate that formed was aged for 24 h at room temperature, and then dried in an air oven for 14 h, at 80 °C. Then, 1 g of hydroxyapatite powder was pressed into a pellet. Sintering of several pellets was carried out at a temperature of 1000 °C with a furnace ramped rate of 5 °C/min and soaking hour within 2 h to form sinter hydroxyapatite scaffolds. Meanwhile, the rest of the pellets that do not undergo sintering process were considered as nonsinter scaffolds.

#### 4.1.1. Field Emission Scanning Electron Microscopy (FESEM)

Pore size analysis was conducted by FESEM micrographs (FESEM) (Carl Zeiss, Germany). Scaffolds were dehydrated using a series of ethanol solution (30, 50, 70, 90, and 100%) for 10 min twice for each ethanol concentration in a critical point dryer machine (CPD) (BAL-TECH CPD 030, USA). The dried samples were sputter coated with gold in order to obtain sufficient conductivity on the surface and to avoid surface charging during the process. Samples were, then, examined under field emission scanning electron microscope. 

#### 4.1.2. Micro-Computed Tomography (microCT)

Porosity of both sinter and nonsinter scaffolds were investigated using a microCT machine (Skyscan, Bruker, Belgium) with the following parameters: 8.88 µm of pixel size, voltage 80 kV, and 118 µA. Three-dimensional constructed images were used to further analyze the porosity using the CTAn analysis software (Bruker, Belgium) at region of interest volume (ROI).

### 4.2. Cell Culture Procedures

The MC3T3-E1 subclone C14 (MC3T3-E1/C14) preosteoblast cell line (ATCC No: CRL-2596™) was used in this study. These cells were cultured in 75 cm2 flasks and maintained in alpha minimal essential medium (αMEM) (Gibco, Grand Island, NY, USA) supplemented with 15% (*v/v*) fetal bovine serum (FBS) (Gibco, Grand Island, NY, USA), 1% (*v/v*) penicillin/streptomycin (Gibco, Grand Island, NY, USA), and 1 mM sodium pyruvate (Sigma Aldrich, St. Louis, MO, USA,). The flasks were incubated at 37 °C, 5% CO_2_, and 95% relative humidity. The medium was changed every three days.

The confluent cells were subcultured through the trypsinization method. When the cells reached 90% confluency, the flask was washed with phosphate buffer saline (PBS) (Gibco, Grand Island, NY, USA) and 1.0 mL of 0.25% (*v/v*) trypsin-EDTA (Gibco, Grand Island, NY, USA) was added to detach the MC3T3-E1 from the flask. After 3 min of incubation, 1.0 mL of complete medium was added to inhibit trypsin activity and the dissociated cells were, then, collected and transferred into 15 mL sterile disposable centrifuge tubes for centrifugation (Eppendorf, Hamburg, Germany) at 1200× *g* for 10 min. The supernatant was removed, and the pellet was resuspended in 1 mL complete medium. The number of viable cells was determined using trypan blue exclusion assay procedure.

### 4.3. Preparation of Scaffolds

Hydroxyapatite scaffolds were obtained from the Faculty of Science, Universiti Putra Malaysia (UPM). The spherical-shaped scaffolds measuring 12 × 5 mm were obtained in two groups, i.e., sinter and nonsinter scaffolds.

Sinter and nonsinter hydroxyapatite scaffolds were autoclaved (120 °C, 20 min) and placed in 24-well plates. Both scaffolds were immersed in complete medium and incubated overnight prior to cell seeding. MC3T3-E1 preosteoblast cells were seeded at 5 × 10^4^/cm^2^ seeding density. Afterwards, fresh medium was added until it reached a final volume of 1 mL per well. The preosteoblast cells were cultured on both hydroxyapatite scaffolds for 7, 14, and 21 days. Complete medium without scaffolds was considered as the control group.

#### 4.3.1. Field Emission Scanning Electron Microscopy (FESEM)

The cells/scaffolds co-cultures were fixed in 2.5% *v/v* glutaraldehyde for 24 h. Then, scaffolds were kept in phosphate buffer saline (PBS) followed by dehydration in serial diluted ethanol using critical point dryer machine. Then, samples were sputter coated with gold for SEM analysis. Then, samples were examined under field emission scanning electron microscope (FESEM) (Carl Zeiss, Munich, Germany).

#### 4.3.2. MTT Assay

Cell viability of MC3T3-E1 preosteoblast cells seeded on scaffolds were determined using MTT assay at day 7, 14, and 21. A group of preosteoblast cell lines without a scaffold was used as the control group. At each timepoint, 1 mL of reconstitute methyl thiazolyl tetrazolium (MTT) reagent was added to each well and incubated for 4 h. The samples were removed from the well after the formation of formazan crystal and 1 mL of DMSO and glycine buffer (ratio 9:1) was added in each well and subjected to ultrasonic stirrer for 1 h. Then, 100 μL of sample from each well were transferred to 96-well plate and the absorbance was measured at 570 nm using ELISA automated plate reader (Bio RAD, Model 680, USA). All experiments were done 3 times independently with sample (*n* = 3).

#### 4.3.3. ALP Assay

The alkaline phosphatase (ALP) assay was performed with MC3T3-E1 preosteoblast cells cultured on both sinter and nonsinter scaffolds for 7, 14, and 21 days. The preosteoblast cells cultured without scaffold were used as the control group. All cells were cultured with complete medium supplemented with 100 μM ascorbic acid and 10 mM β-glycerophosphate. The assay was conducted using a Sensolyte^®^ pNPP alkaline phosphatase assay kit (AnaSpec, USA). Cells were detached from scaffolds in a 500 μL lysis buffer, provided in the kit, for 10 min. Lysates were centrifuged at 2500× *g* at 4 °C for 10 min. Supernatant was collected for the ALP assay using p-nitrophenyl phosphate (pNPP) as substrate. Each specimen was incubated with the addition of 100 μL of a pNPP solution at 37 °C for 30 min. The production of p-nitrophenol in the presence of ALP was measured by monitoring light absorbance by solution at 405 nm using ELISA reader (Bio RAD, Model 680, CA, USA), as per manufacturer’s instruction. The results were normalized to the amount of ALP provided in the kit as a standard.

#### 4.3.4. Real-Time Polymerase Chain Reaction (rtPCR)

The expression of osteogenesis-related genes were quantified using osteocalcin (OCN) and Type I collagen (Col1α1) as osteoblastic marker; while Gapdh was used as housekeeping genes in this study.

The cells were seeded at the same density of 5 × 104 cells/cm^2^ on scaffolds in 24-well plates. Cells were cultured for 14 days, and then harvested to extract RNA. On day 14, cells from the cells/scaffold co-culture were extracted using TRIzol reagent. Following that, cell lysate from the aqueous phase formed during the separation phase were used for further steps. RNA was extracted from lysate using an innuPREP RNA kit (Analytikjena, Jena, Germany), according to the manufacturer’s instruction. The total RNA was, then, quantified using a Nanodrop 2000 (Thermo Scientific, Waltham, MA, USA) at 260 and 280 nm wavelength. The purity of each sample was identified using A260/A280 ratio. A ratio of 1.8 to 2.0 was considered to be purified RNA sample. Then, extracted RNA was stored at −80 °C until further molecular analysis.

Complementary DNA (cDNA) synthesis was performed using a commercially cDNA synthesis kit (Bioline, Memphis, USA). 1 μL of extracted RNA was reverse transcribed by using reverse transcriptase (RT) to generate full-length first strand cDNA from the total RNA extracted from the preosteoblast cells in a Thermal Cycler (BioRad, CA, USA). The RT steps consisted of the primer annealing stage at 25 °C for 10 min, followed by the reverse transcription stage at 42 °C for 15 min and the inactivation stage 85 °C for 5 min. The RT products were used as templates for the polymerase chain reaction (PCR) amplification of 20 μL reactions.

The rt-PCR analysis of genes included type I collagen (col1a1) and osteocalcin (OCN), performed using a Sybr Green Kit (Bioline, USA). The primers for the target genes are listed in Table 1. The primer sequences for rt-PCR were designed using Primer-BLAST. The synthesized cDNA was used as the template for polymerase chain reaction (PCR) amplification to study gene expression in these cells. A real-time polymerized chain reaction (rt-PCR) was performed with 10 μL of SYBR Green Mix, 1 μL of each target genes forward and backward primer, 1 μL of cDNA template, and 7 μL of dH2O. The mixture was incubated at 95 °C for 5 s (denaturation stage) and 58 °C for 10 s for 40 cycles (annealing stage), terminating at 72 °C for 10 s. The Ct values of target genes were normalized by the Ct values of the Sybr Green human housekeeping gene (Gapdh) to obtain the 2ΔCt values.

### 4.4. Statistical Analysis

Data were presented as the average of biological and technical replicates (mean ± standard deviation). Statistical analysis was carried out with two-way mixed analysis of variance (ANOVA) using the Statistical Package for the Social Sciences (SPSS) version 23. A further follow-up comparison between groups was conducted with one-way ANOVA and (*p* < 0.05) was accepted as statistically significant difference.

## 5. Conclusions

In this study, sinter nacre showed better physical and bioactivity as compared with the nonsinter group, as a potential biomaterial for bone tissue engineering. Nacre-derived hydroxyapatite scaffolds that underwent the sintering process prior would be a promising ceramic material in dentistry. Because bone defects are among diseases that are significant in dentistry, bone regeneration would potentially become an alternative option among surgeons. The less invasive approach of tissue engineering as compared with autologous grafting has significant advantages for reducing complications from the latter method, especially in children. Although the current findings of using the sintering process are promising, further in vivo and clinical studies are required to demonstrate the superior bioactivity and novel properties of nacre for bone regeneration.

## Figures and Tables

**Table 1:** Primer sequences for target genes.

Target Genes	Primer Sequences		Product Size (bp)
OCN	GGCCCTGAGTCTGACAAAGC	Forward	21
	GCCGGAGTCGTTCACTACCTT	Reverse	20
Col1α1	ATGGATTCCCGTTCGAGTACG	Forward	20
	TCAGCTGGATAGCGACATCG	Reverse	22
Gapdh	ATGTGTCCGTCGTGGATCTGA	Forward	21
	TTGAAGTCGCAGGAGACAACCT	Reverse	22
